# Mineralocorticoid Receptor Signaling Contributes to Normal Muscle Repair After Acute Injury

**DOI:** 10.3389/fphys.2019.01324

**Published:** 2019-10-25

**Authors:** J. Spencer Hauck, Zachary M. Howard, Jeovanna Lowe, Neha Rastogi, Madison G. Pico, Sarah A. Swager, Jennifer M. Petrosino, Celso E. Gomez-Sanchez, Elise P. Gomez-Sanchez, Federica Accornero, Jill A. Rafael-Fortney

**Affiliations:** ^1^Department of Physiology and Cell Biology, College of Medicine, The Ohio State University, Columbus, OH, United States; ^2^Dorothy M. Davis Heart and Lung Research Institute, College of Medicine, The Ohio State University, Columbus, OH, United States; ^3^Department of Internal Medicine, University of Mississippi Medical Center, Jackson, MS, United States; ^4^Department of Pharmacology and Toxicology, University of Mississippi Medical Center, Jackson, MS, United States

**Keywords:** mineralocorticoid receptor, mineralocorticoid receptor antagonist, conditional knockout mouse, spironolactone, myofiber, muscle injury

## Abstract

Acute skeletal muscle injury is followed by a temporal response of immune cells, fibroblasts, and muscle progenitor cells within the muscle microenvironment to restore function. These same cell types are repeatedly activated in muscular dystrophy from chronic muscle injury, but eventually, the regenerative portion of the cycle is disrupted and fibrosis replaces degenerated muscle fibers. Mineralocorticoid receptor (MR) antagonist drugs have been demonstrated to increase skeletal muscle function, decrease fibrosis, and directly improve membrane integrity in muscular dystrophy mice, and therefore are being tested clinically. Conditional knockout of MR from muscle fibers in muscular dystrophy mice also improves skeletal muscle function and decreases fibrosis. The mechanism of efficacy likely results from blocking MR signaling by its endogenous agonist aldosterone, being produced at high local levels in regions of muscle damage by infiltrating myeloid cells. Since chronic and acute injuries share the same cellular processes to regenerate muscle, and MR antagonists are clinically used for a wide variety of conditions, it is crucial to define the role of MR signaling in normal muscle repair after injury. In this study, we performed acute injuries using barium chloride injections into *tibialis anterior* muscles both in myofiber MR conditional knockout mice on a wild-type background (MRcko) and in MR antagonist-treated wild-type mice. Steps of the muscle regeneration response were analyzed at 1, 4, 7, or 14 days after injury. Presence of the aldosterone synthase enzyme was also assessed during the injury repair process. We show for the first time aldosterone synthase localization in infiltrating immune cells of normal skeletal muscle after acute injury. MRcko mice had an increased muscle area infiltrated by aldosterone synthase positive myeloid cells compared to control injured animals. Both MRcko and MR antagonist treatment stabilized damaged myofibers and increased collagen infiltration or compaction at 4 days post-injury. MR antagonist treatment also led to reduced myofiber size at 7 and 14 days post-injury. These data support that MR signaling contributes to the normal muscle repair process following acute injury. MR antagonist treatment delays muscle fiber growth, so temporary discontinuation of these drugs after a severe muscle injury could be considered.

## Introduction

Skeletal muscle comprises up to 40% of total body mass and is responsible for breathing, posture, and locomotion in mammals (Holmberg and Durbeej, 2013; Jarvinen et al., 2013; Yin et al., 2013; Frontera and Ochala, 2015; Joanisse et al., 2017). Since skeletal muscle is so abundant, it often becomes damaged through a single traumatic event, such as laceration, sports, car collision, combat, or extreme temperature (Jarvinen et al., 2013; Wall et al., 2015; Hardy et al., 2016). As a result, muscle function is reduced until the muscle recovers from injury (Lu et al., 2011; Jarvinen et al., 2013; Souza and Gottfried, 2013; Wall et al., 2015; Hardy et al., 2016). After acute muscle injury, many cell types within the skeletal muscle microenvironment must respond sequentially to mitigate damage and regenerate myofibers, the cell type responsible for muscle contraction (Bentzinger et al., 2013; Juban and Chazaud, 2017; Tidball, 2017; Cretoiu et al., 2018). Myeloid immune cell infiltration peaks at 2–3 days post-injury in mice to remove cellular debris resulting from damaged myofibers (Bentzinger et al., 2013; Juban and Chazaud, 2017; Tidball, 2017). Fibroblasts proliferate approximately 4 days post-injury in mice and secrete extracellular matrix proteins including collagen to stabilize the surrounding undamaged myofibers (Bentzinger et al., 2013; Juban and Chazaud, 2017; Tidball, 2017). From 2 to 5 days post-injury in mice, muscle progenitor cells nucleate new myofibers containing centrally located nuclei (Bentzinger et al., 2013; Hardy et al., 2016; Tidball, 2017). By 7 days post-injury, all new myofibers have been formed and will grow in size and mature until the muscle is completely recovered by about a month post-injury (Bentzinger et al., 2013; Hardy et al., 2016; Tidball, 2017). Myogenic factors are responsible for driving the muscle regeneration response (Rodriguez et al., 2014; Sharples et al., 2015; Hernandez-Hernandez et al., 2017). The ability to decrease skeletal muscle damage and/or expedite muscle repair would reduce recovery time after injury.

In contrast to acute muscle injury, chronic muscle injury diseases including Duchenne muscular dystrophy involve repeated damage to skeletal muscle (Bentzinger et al., 2013; Tidball et al., 2018). The muscle microenvironment in chronic muscle injury attempts to compensate for the repeated damage and regenerate muscle in a similar manner to acute muscle injury (Bentzinger et al., 2013; Tidball et al., 2018). The mineralocorticoid receptor (MR) has been identified as an important therapeutic target for modifying disease severity in Duchenne muscular dystrophy (Duboc et al., 2005; Rafael-Fortney et al., 2011, 2016; Sayer and Bhat, 2014; Chadwick et al., 2015, 2017a; Lowe et al., 2016; Raman et al., 2017; Hauck et al., 2019). The MR, a nuclear steroid hormone receptor, is expressed in many cell types in the skeletal muscle microenvironment including myofibers, muscle progenitor cells, immune cells, fibroblasts, and endothelial cells (Duboc et al., 2005; Rickard et al., 2009, 2014; Yang and Young, 2009; Usher et al., 2010; Lother et al., 2011; Chadwick et al., 2015; Mueller et al., 2015; Hauck et al., 2019). Chronic overactivation of MR by the endogenous hormone aldosterone, primarily produced from the adrenal gland, is known to exacerbate cell damage in cardiovascular diseases and promote fibrosis (Mizuno et al., 2001; Pitt, 2012; McCurley et al., 2013; Gomez-Sanchez and Gomez-Sanchez, 2014). We have shown in mouse models of muscular dystrophy that genetic inactivation of myofiber MR improves skeletal muscle force and alters fibrosis (Rafael-Fortney et al., 2011; Lowe et al., 2016; Hauck et al., 2019). Additionally, our findings indicate that MR antagonist treatment improves muscle membrane integrity in muscular dystrophy (Chadwick et al., 2017a; Hauck et al., 2019). We have also demonstrated that myeloid immune cells in dystrophic skeletal muscle contain the enzyme aldosterone synthase (CYP11B2) and have the capacity to synthesize aldosterone (Chadwick et al., 2016). These data suggest that chronic MR signaling from inflammatory cells contribute to pathology in muscular dystrophy. However, acute injury responses often differ from chronic pathological states and the role of MR in the acute setting is completely unknown.

To understand whether MR signaling contributes to normal muscle repair, we investigated the role of MR in the myofiber and the overall muscle microenvironment during acute injury. The results of this study will identify whether MR modulation can be incorporated into the treatment of patients with acute muscle injuries.

## Materials and Methods

### MRcko and Spironolactone-Treated Mice

All protocols were approved by the Institutional Animal Care and Use Committee of The Ohio State University, are in compliance with the laws of The United States of America, and conform to the National Institutes of Health Guide for the Care and Use of Laboratory Animals. Mineralocorticoid receptor (MR) floxed mice on a C57BL6/NCrL background were crossed to hemizygous muscle creatine kinase driven Cre recombinase transgene (MCK-Cre) mice (Jackson Laboratories, Bar Harbor, ME, 006475 mouse line on a C57BL/6 background) to make a myofiber specific conditional knockout (MR^flox/flox^; MCK-Cre+ aka MRcko) and Cre− control littermates (MR^flox/flox^; Cre−) (Berger et al., 1998, 2006; Bruning et al., 1998; Usher et al., 2010; Hauck et al., 2019). Conditional knockout of the MR in myofibers has been validated, and mice were genotyped as previously described (Hauck et al., 2019). To validate excision of the floxed exon of the MR locus specifically in acutely injured *tibialis anterior* (TA), we performed PCR on MRcko genomic DNA. Excision PCR was performed on acutely injured TAs from mice at 4 (*n* = 5 MRcko barium chloride [3M, 2F], *n* = 1 MRcko PBS [M], *n* = 1 Cre− barium chloride [M]) and 7 (*n* = 5 MRcko barium chloride [2M, 3F], *n* = 1 MRcko PBS [M], *n* = 1 Cre− barium chloride [M]) days post-injury as previously described (Hauck et al., 2019). Presence of Cre recombinase results in excision of the MR floxed allele that generates the MR null allele. The excision PCR was quantified with ImageJ (Bethesda, MD) and expressed as injured MRcko mouse TA band intensity/MRcko mouse injected with sterile phosphate buffered saline (PBS) TA at 7 days post-injection. All PCR were run on a ProFlex PCR System (ThermoFisher Scientific, Waltham, MA). C57BL/10 mice were maintained as a separate colony and treated with spironolactone in water bottles as previously described (Rafael-Fortney et al., 2011; Lowe et al., 2018) for 2 weeks prior to barium chloride-induced acute muscle injury and during the days following injury until sacrifice.

### Barium Chloride-Induced Acute Muscle Injury

MRcko and Cre− C57BL6/NCrL control mice of both sexes at 8–10 weeks-of-age were anesthetized with isoflurane and hair on the anterior portion of both lower legs was removed with Baby Oil Nair Lotion (Church and Dwight Co., Ewing, NJ). After Nair treatment, the leg was rinsed well with sterile water, using a non-woven sponge and dried. The mice were injected (Becton Dickinson, Franklin Lakes, NJ, 3/10 cc U-100 Insulin syringe, 30G × 3/8″ needle) intramuscularly into the middle portion of the mouse’s left TA with 50 μl of sterile 1.2% barium chloride (Sigma-Aldrich, St. Louis, MO, B0750) diluted in sterile water as previously described (Dekeyser et al., 2013; Singhal and Martin, 2015; Hardy et al., 2016). To serve as a control, the right TA muscle was injected with 50 μl of sterile saline. Mice were sacrificed at 1, 4, 7, or 14 days post-injury. The same procedure was also performed on spironolactone treated and untreated C57BL/10 control mice of both sexes at 8–10 weeks-of-age. MRcko and Cre− littermates from litters born within the 2 week-range were injected together and aged to one of the analyzed time points. The injections for analysis at different time points were not done at the same time due to technical feasibility to obtain the required numbers of mice. Obtaining required numbers of experimental and control mice for analysis at each time point typically required injections of two separate groups of littermates and sometimes as many as three groups. Similarly, spironolactone and untreated C57BL/10 control littermates from litters born within the 2 week-range were injected together and aged to one of the analyzed time-points, but these experiments were performed after the completion of the MRcko and Cre− analyses. Therefore, pairwise statistical analyses were performed only comparing MRcko and Cre− littermate controls that were injected for analysis at the same time point. The statistical analysis for spironolactone and untreated C57BL/10 controls was handled in the same way.

### Histological Staining and Analysis

Mouse TA muscles were dissected from the mice and cut at midbelly; half was snap frozen in liquid nitrogen and half was embedded in optimal cutting compound. The embedded TA muscles were frozen in liquid nitrogen-cooled isopentane and later sectioned at a thickness of 8 μm. Any section containing an excess of longitudinal skeletal muscle or had less than 50% centrally nucleated myofibers was excluded from analysis. All immunofluorescence sections were mounted with Vectashield mounting medium with 2 ng/μl of DAPI to visualize nuclei.

#### CYP11B2 Immunohistochemistry

To evaluate levels of CYP11B2 in acutely injured muscle, immunohistochemistry was performed on muscle from mice sacrificed at 1 (*n* = 3 Cre− [1M, 2F]), 4 (*n* = 3 Cre− [3F]), and 7 (*n* = 3 Cre− [3 M]) days after injury. The sections were incubated with: 1:250 CYP11B2 (rabbit anti-mouse AS 2084) and then incubated with 1:200 HRP-conjugated goat anti-rabbit IgG (Jackson Immuno Research Laboratories, West Grove, PA, 111-035-144) and developed with ImmPact DAB peroxidase substrate (Vector, Burlingame, CA, SK-4105). Since 4 days appeared to have the highest levels of CYP11B2, additional 4 days post-injury sections were analyzed (*n* = 6 Cre− [2M, 4F], and 6 MRcko [3M, 3F]) (*n* = 6 untreated [3M, 3F], and 6 spironolactone [3M, 3F]). Images of the CYP11B2 staining were taken on a Nikon (Melville, NY) Eclipse 800 microscope with the 20× objective and ND32 and NCB11 filters with the SPOT digital camera software and composited using Adobe Photoshop (Adobe, San Jose, CA, CS6). The percent area of CYP11B2 infiltration was quantified with the Photoshop (Adobe, San Jose, CA, CS6) paint bucket tool by an individual blinded to the genotypes and treatment as previously described (Hauck et al., 2019).

#### CYP11B2 and CD11b Co-immunostaining

To determine if CYP11B2 was present in CD11b^+^ monocytes and macrophages in acutely injured muscle, Cre− sections were incubated with: 1:500 CYP11B2 (rabbit anti-mouse AS 2084) along with the 1:50 CD11b (rat anti-mouse monoclonal BD Pharmingen, San Jose, CA, 550282) primary antibodies and then incubated with 1:200 Cy3-conjugated goat anti-rat IgG (Jackson Immuno Research Laboratories, West Grove, PA, 112-165-167) along with 1:200 Alexa Fluor R 555 conjugated goat anti-rabbit antibody (Invitrogen, Waltham, MA, A21429) (*n* = 4 Cre− [1M, 3F]) (Chadwick et al., 2016). Pictures were taken on a Nikon (Melville, NY) Eclipse 800 microscope with the SPOT RT slider digital camera software.

#### Quantification of Ongoing Degeneration

For quantification of the number of actively degenerating myofibers, MRcko 4 days post-injury sections were incubated with: 1:200 Alexa Fluor R 488 conjugated goat anti-mouse IgG antibody (Invitrogen, Waltham, MA, A11029) (*n* = 15 Cre− [7M, 8F], and 13 MRcko [8M, 5F]). An individual blinded to the genotypes counted the number of IgG positive fibers under a Nikon (Melville, NY) Eclipse 800 microscope with the 20× objective. For quantification of degenerating myofibers, spironolactone-treated 4 days post-injury sections were quantified and analyzed as previously described (*n* = 16 untreated [7M, 9F], and 16 spironolactone [9M, 7F]) (Hauck et al., 2019).

#### Central Nuclei Percentage and Myofiber Size

To measure percent of centrally nucleated myofibers and centrally nucleated myofiber size in MRcko mice, the sections were stained with: 1:500 laminin-2 alpha 2 chain (rat anti-mouse monoclonal Sigma-Aldrich, St. Louis, MO, L0663) primary antibody and then incubated with 1:200 Cy3-conjugated goat anti-rat IgG secondary antibody (Jackson Immuno Research Laboratories, West Grove, PA, 112–165-167) at 4 (*n* = 15 Cre− [7M, 8F], and 13 MRcko [8M, 5F]), 7 (*n* = 12 Cre− [7M, 5F], and 14 MRcko [6M, 8F]), and 14 (*n* = 11 Cre− [3M, 8F], and 13 MRcko [5M, 8F]) days post-injury. Images were taken on a Nikon (Melville, NY) Eclipse 800 microscope with the 10× objective with a SPOT RT slider digital camera software and composited using Adobe Photoshop (Adobe, San Jose, CA, CS6). To measure percent of centrally nucleated myofibers and centrally nucleated myofiber size in spironolactone-treated mice, the spironolactone degenerating myofibers sections were used and analyzed as previously described at 4 (*n* = 9 untreated [2M, 7F], and 8 spironolactone [4M, 4F]), 7 (*n* = 8 untreated [5M, 3F], and 10 spironolactone [6M, 4F]), and 14 (*n* = 12 untreated [5M, 7F], and 13 spironolactone [7M, 6F]) days after acute muscle injury (Hauck et al., 2019). Myofiber size for both MRcko and spironolactone-treated mice along with the respective control was measured with the semi-automatic muscle analysis using segmentation of histology (SMASH) MatLab (Mathworks, Natick, MA) plug-in as previously described except a myofiber maximum size of 3,600 μm^2^ was used instead of 5,700 μm^2^ because the TA myofibers are not as large a quadriceps myofibers (Smith and Barton, 2014; Hauck et al., 2019).

#### Immune Cells, Fibroblasts, and Collagen Infiltration

For the immune cell and fibroblast infiltration analysis, the sections at 4 days post-injury were stained with CD11b and vimentin antibodies and quantified as previously described (*n* = 16 untreated [7M, 9F], and 16 spironolactone [9M, 7F]) (Hauck et al., 2019). For collagen analysis, sections at 4 days post-injury were stained with picrosirius red, imaged under brightfield and polarized light, and quantified (Polysciences, Warrington, PA, 24901-500) as previously described (Hauck et al., 2019) (*n* = 7 Cre− [3M, 4F], and 8 MRcko [4M, 4F]) (*n* = 9 untreated [2M, 7F], and 8 spironolactone [4M, 4F]) (Hauck et al., 2019; Petrosino et al., 2019).

### Quantitative Polymerase Chain Reaction Analysis

For myogenic factor and growth control transcript analysis, spironolactone-treated mouse TA at 7 days post-injury was compared to untreated (*n* = 5 untreated [3M, 2F], and 5 spironolactone [3M, 2F]) for transcript levels of myogenic markers myostatin, insulin-like growth factor 1, myogenin, and myoblast determination protein 1 as previously described with primers listed in Table 1 (Chadwick et al., 2015; Hauck et al., 2019). For collagen transcript analysis, spironolactone-treated mouse TA at 4 days post-injury was analyzed (*n* = 5 untreated [2M, 3F], and 5 spironolactone [3M, 2F]) for transcript levels of collagens type 1, 3, 8, and 11 with quantitative polymerase chain reaction (qPCR) as previously described with primers listed in Table 1 (Chadwick et al., 2015; Hauck et al., 2019).

**Table 1:** Primers used for qPCR analyses.

Transcript	Primers	Product size (bp)
*Mstn*	mMstn For 5′-GCACTGGTATTTGGCAGAGT-3′ mMstn Rev 5′-TCAAGCCCAAAGTCTCTCCG-3′	217
*Igf1*	mIgf1 For 5′-CGCAATGGAATAAAGTCCTCAAAAT-3′ mIgf1 For 5′- CCAGGTAGAAGAGGTGTGAAGA-3′	234
*Myog*	mMyog For 5′-GCCATCCAGTACATTGAGCG-3′ mMyog Rev 5′-GTTGGGACCGAACTCCAGTG-3′	174
*Myod1*	mMyod1 For 5′-ATCCGCTACATCGAAGGTCTG-3′ mMyod1 Rev 5′-GGTGTCGTAGCCATTCTGCC-3′	222
*Col1a1*	mCol1A1 For 5′-AGGGTCATCGTGGCTTCTCT-3′ mCol1A1 Rev 5′-GGAGACCGTTGAGTCCGTC-3′	153
*Col3a1*	mCol3A1 For 5′-TCCACGAGGTGACAAAGGTG-3′ mCol3A1 Rev 5′-CTGGATGCCCACTTGTTCCA-3′	206
*Col8a1*	mCol8A1 For 5′-TGGCAAAGAGTACCCACACC-3′ mCol8A1 Rev 5′-CAGGCATTCCATGACCTGGT-3′	197
*Col11a1*	mCol11A1 For 5′-CTCACCATCTCAACCCTCGC-3′ mCol11A1 Rev 5′-GCTGATTTGTGCTTCCTCCG-3′	201

### Statistical Analysis

Group sizes for each assay were based on previous results in MRcko mice on a muscular dystrophy background (Hauck et al., 2019). All data for each analysis and at each time point were included in statistical calculations except for primarily longitudinal sections and sections that had less than 50% centrally nucleated myofibers to exclude samples without sufficient damage from the acute muscle injury. Injections for analysis at different time points and for the genetic (MRcko) and pharmacological (spironolactone) experiments were not done at the same time due to technical feasibility to obtain the required numbers of mice. Therefore, data for experimental mice were compared only to data from the control group at the same time point for each the genetic and pharmacological experiments using a Student’s *t*-test assuming equal variance. The only exception is that 7 days post-BaCl2 was compared to 4 days post-BaCl2 for the excision PCR in Figure 2. Values from the left TA of each mouse were used for all statistical analyses, except for the excision PCR when the left TA values were expressed as a percentage of 7 day right TA values. Summary values are presented as mean ± standard error. Males and females were also analyzed separately, but there appeared to be no substantial differences by sex for the parameters analyzed.

## Results

### CYP11B2 Is Present in Normal Skeletal Muscle After Acute Injury

To define whether MR signaling contributes to normal muscle damage and recovery from acute injury, we used mice with a conditional myofiber MR knockout (MRcko) where excision of the MR “floxed” allele is driven by a muscle creatine kinase regulated Cre recombinase (Hauck et al., 2019). MRcko and Cre− wild-type control littermates (MR^flox/flox^; Cre−) were acutely injured by injecting barium chloride into the left *tibialis anterior* (TA) muscle, with phosphate buffered saline (PBS) injection of right TAs serving as contralateral controls. Barium chloride intramuscular injection causes severe skeletal muscle injury through hypercontraction and was chosen for acute muscle injury due to its relatively low overall toxicity and reproducibility (Karaki et al., 1986; DalleDonne et al., 1998; Guess et al., 2015; Hardy et al., 2016). Barium chloride was selected over cardiotoxin for acute injury because cardiotoxin has batch to batch variability and indirectly causes muscle hypercontraction through inhibition of protein kinase C (Charge and Rudnicki, 2004; Souza and Gottfried, 2013; Hardy et al., 2016).

**Figure 1:**
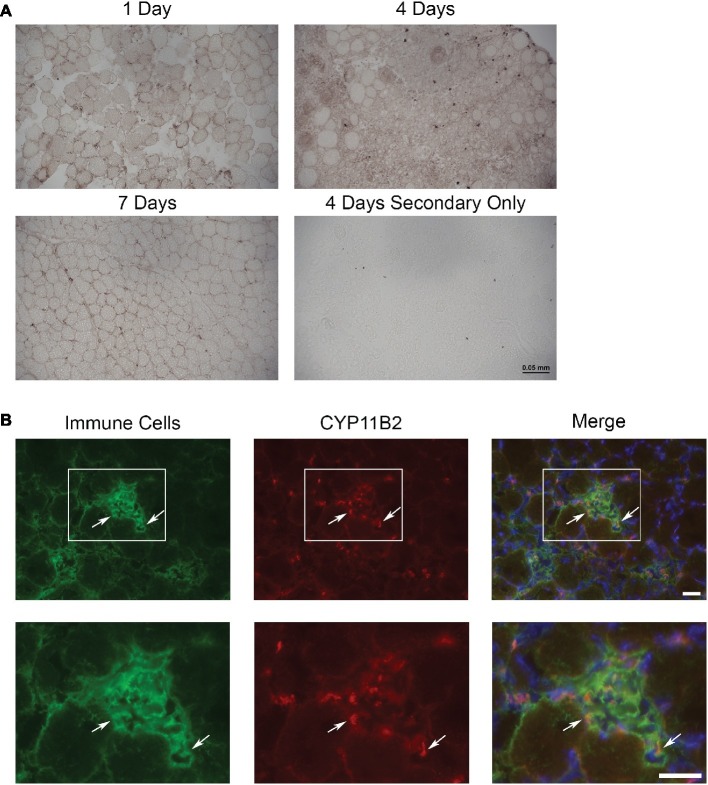
CYP11B2 is present in acutely injured skeletal muscle. **(A)** The presence of CYP11B2 aldosterone synthase at 1, 4, and 7 days after acute muscle injury in *tibialis anterior* of wild-type mice was evaluated with immunohistochemistry (brown) and appeared to be present at the highest level at 4 days post-injury (*n* = 3 Cre− mice per time point). Scale bar = 50 μm. **(B)** CYP11B2 (red) co-localized (arrows) with CD11b^+^ immune cells (green) at 4 days post-injury (*n* = 4 Cre−) similar to that observed in chronic muscle injury (Chadwick et al., 2016). DAPI was used to stain nuclei (blue) in the merged image. The co-localization of CYP11B2 and CD11b^+^ immune cells can be better observed in the zoomed images (bottom panels) for each channel from the area represented by the white box in the top panels. Scale bars = 25 μm.

To determine if aldosterone synthase (CYP11B2) was present in acutely injured muscles, we evaluated the TAs of Cre− mice at 1, 4, and 7 days post-injury using immunohistochemistry with an antibody against CYP11B2 (Figure 1A). Of the time points evaluated, CYP11B2 showed the most prevalent staining at 4 days post-injury, which correlates with the timing of immune cell infiltration into skeletal muscle (Bentzinger et al., 2013; Juban and Chazaud, 2017; Tidball, 2017). To determine if immune cells contain CYP11B2 in acute muscle injury, we co-stained TA muscles of Cre− mice at 4 days post-injury with CYP11B2 and the myeloid immune cell marker CD11b (Chadwick et al., 2016). The CYP11B2 staining localized to CD11b^+^ cells (Figure 1B).

### Newly Formed Myofibers Have High Levels of MR Excision by 7 Days Post-injury

**Figure 2:**
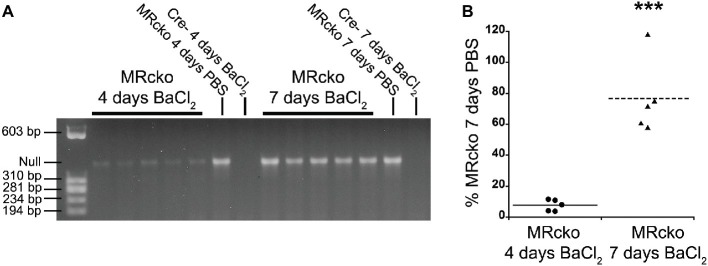
Evaluation of MRcko excision after acute muscle injury. **(A)** PCR was run to detect the level of MR excision by presence of the MR null allele in TAs from mice at 4 (*n* = 5 MRcko barium chloride injection, *n* = 1 MRcko PBS injection, *n* = 1 Cre− barium chloride injection) and 7 (*n* = 5 MRcko barium chloride injection, *n* = 1 MRcko PBS injection, *n* = 1 Cre− barium chloride injection) (two technical replicates) days post-injury. **(B)** The level of MR null allele at 4 and 7 days post-injury was quantified and normalized to the level of MR null allele from PBS injection at 7 days post-injury. There was restoration of MR excision at 7 days post-injury (*p* = 0.0002). Means are shown by lines for each group in the dot plot. BaCl_2_, barium chloride injection; PBS, sterile phosphate buffered saline injection; TA, *tibialis anterior*; data were analyzed using a Student’s *t*-test; ^***^p ≤ 0.001 compared to MRcko barium chloride injection at 4 days post-injury.

We have previously defined efficient myofiber MR knockout in MRcko mice at baseline (Hauck et al., 2019). We next sought to define the time point when MRcko disrupts aldosterone signaling to newly regenerated myofibers. Since the muscle creatine kinase promoter only drives Cre expression in differentiated myofibers, we evaluated excision of the MR floxed allele by Cre recombinase in MRcko mouse TA muscles at 4 and 7 days post-injury in comparison to MRcko mouse right TA contralateral control muscles (Figure 2A). PCR of genomic DNA isolated from left TAs identified low levels of MR excision at 4 days post-injury (7.64 ± 1.65% relative to MRcko mouse right TA at 7 days post-injection) (Figures 2A,B). The MR null allele was present at a high level in MRcko mouse right TA at 4 days post-injection (83.74% relative to MRcko mouse right TA at 7 days post-injection) as predicted due to the small amount of damage and presence of newly regenerated fibers after injection of saline. However, by 7 days post-injury, the MR null allele was present at a high level (76.73 ± 10.83% relative to MRcko mouse right TA 7 days post-injection) compared to MRcko left TAs at 4 days post-injury (*p* = 0.0002) (Figures 2A,B). These data are consistent with the timing of muscle creatine kinase driven conditional knockout in mature myofibers after cardiotoxin injury (Bi et al., 2016). Any observed phenotype in MRcko mice from acute muscle injury is from myofiber MR conditional knockout at the time of injury or at 7 days post-injury when newly differentiated myofibers have sufficient expression of the muscle creatine kinase driver of Cre recombinase to excise the MR floxed allele.

### MRcko and Mineralocorticoid Receptor Antagonist Drug Treatment Increase the Number of Damaged Fibers 4 Days Post-injury

The effect of MR signaling on muscle damage and myofiber growth after acute muscle injury was evaluated at key time points after injury. Four days post-injury was chosen to analyze myofiber damage and fibrosis (Bentzinger et al., 2013;Hardy et al., 2016;Juban and Chazaud, 2017;Tidball, 2017). Seven and fourteen days post-injury was chosen to evaluate newly formed regenerating myofibers initially after formation and after growth, respectively (Hardy et al., 2016; Juban and Chazaud, 2017; Tidball, 2017). Since MRcko mice only allow evaluation of MR signaling in mature myofibers, we also sought to more globally assess the results of MR inhibition after acute injury. Therefore, to evaluate the contribution of MR signaling in all cell types present in the entire muscle microenvironment throughout the injury and repair process, we also evaluated outcomes after acute injury in wild-type mice treated with the MR antagonist spironolactone.

**Figure 3:**
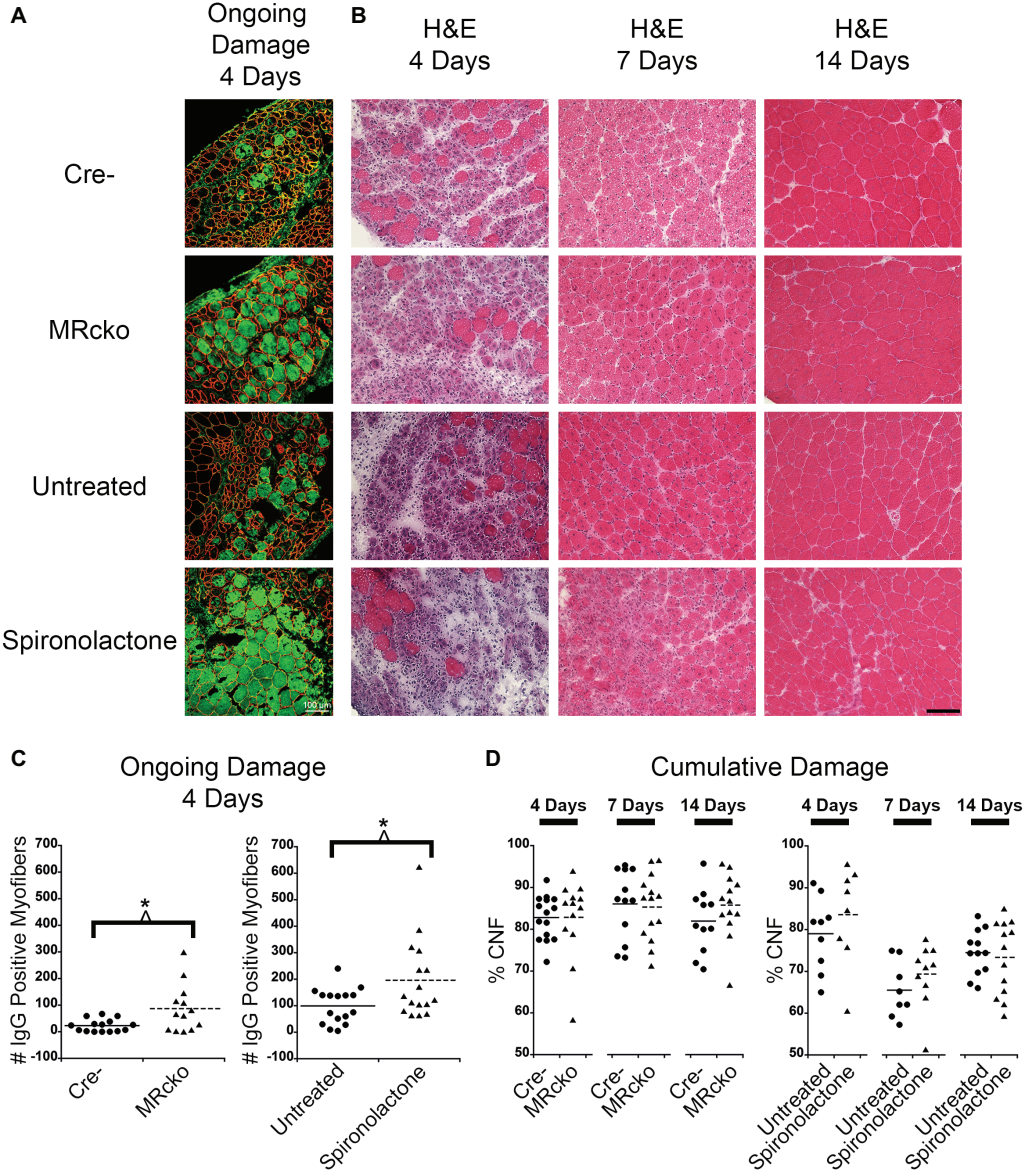
Increased percentages of degenerating myofibers in both MRcko and MR antagonist-treated wild-type mice. **(A)** Representative confocal images of IgG and laminin staining of 4 days post-injury *tibialis anterior* (TA) sections. Scale bar = 100 μm. **(B)** Representative TA sections at 4, 7, and 14 days post-injury stained with hematoxylin and eosin. Scale bar = 100 μm. **(C)** Dot plots showing number of IgG positive fibers at 4 days post-injury for MRcko and Cre− littermates (*n* = 15 Cre−, and 13 MRcko) and spironolactone treated and untreated wild-type littermate mice (*n* = 16 untreated, and 16 spironolactone). Means are shown by lines for each group in the dot plot. **(D)** Dot plots showing percent of myofibers with centralized nuclei in MRcko mice and control littermates at 4 (*n* = 15 Cre−, and 13 MRcko), 7 (*n* = 12 Cre−, and 14 MRcko), and 14 (*n* = 11 Cre−, and 13 MRcko) days post-injury and spironolactone-treated mice at 4 (*n* = 9 untreated, and 8 spironolactone), 7 (*n* = 8 untreated, and 10 spironolactone), and 14 (*n* = 12 untreated, and 13 spironolactone) days post-injury. Means are shown by lines for each group. H & E, hematoxylin and eosin; data were analyzed using a Student’s *t*-test; ^*^p ≤ 0.05.

Spironolactone treatment was initiated 2 weeks prior to injection of barium chloride based on previously observed gene expression changes in this time period (Chadwick et al., 2015). Treatment was continued until sacrifice at each of the time points. To determine if MRcko and/or spironolactone treatment affected myofiber stability, we quantified the number of damaged myofibers in TAs at 4 days post-injury using immunofluorescence for serum IgG. We observed an increase (*p* = 0.0135) in the number of damaged myofibers at 4 days post-injury for MRcko mice (87 ± 25) compared to Cre− (23 ± 6) littermates. There was also an increase (*p* = 0.0273) in damaged myofibers at 4 days post-injury in spironolactone-treated mice (196 ± 38) compared to untreated littermates (99 ± 17) (Figures 3A,C). Overall muscle damage in hematoxylin and eosin stained TA sections supported that MRcko mice and spironolactone treatment increased the persistence of muscle damage at 4 days post-injury (Figure 3B). However, there was no difference in the number of regenerated myofibers between MRcko and Cre− littermates or between spironolactone and untreated littermates at any time point post-injury as assessed by the percentage of centrally nucleated myofibers signifying the cumulative damage incurred by the muscle (Figure 3D). By 7 days post-injury, gross morphology of TA sections supported that efficient myofiber regeneration had occurred in MRcko, Cre−, and untreated wild-type mice (Figure 3B). However, TAs from spironolactone-treated mice at 7 days post-injury still had obvious mononuclear cell infiltrate, fibrosis, and small newly formed myofibers compared to the other groups of mice. Moreover, the TAs from spironolactone-treated mice at 14 days post-injury appeared to have small newly formed myofibers compared to untreated controls (Figure 3B).

### Spironolactone Treatment Delays Myofiber Growth After Acute Muscle Injury

**Figure 4:**
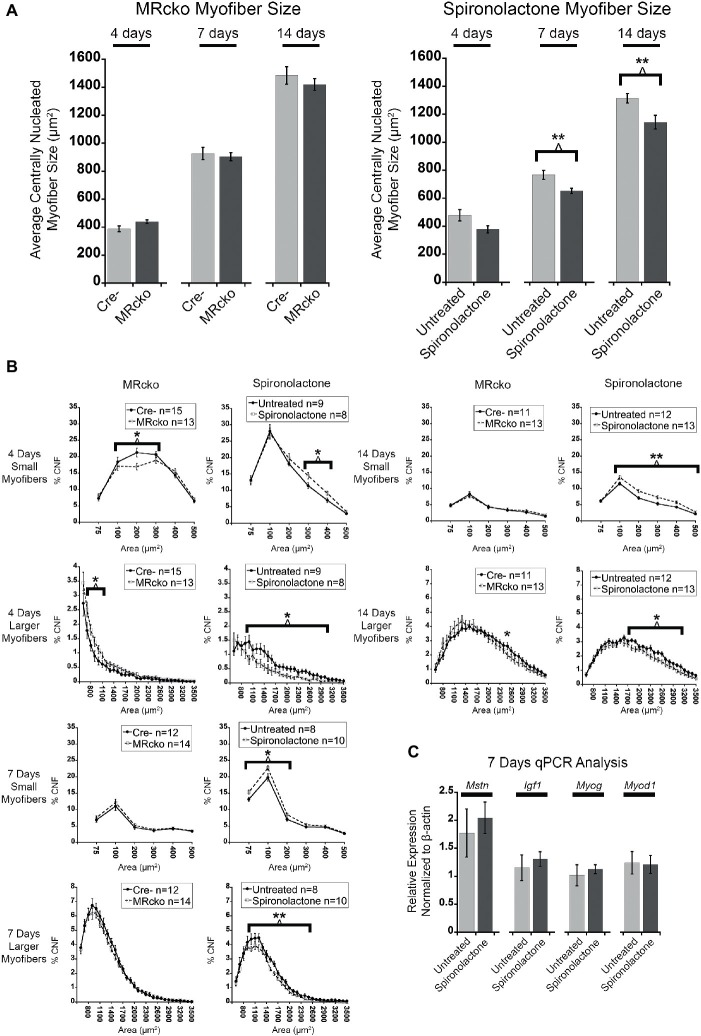
MR antagonism reduces myofiber size after acute muscle injury. **(A)** Bar graphs showing the average size of centrally nucleated *tibialis anterior* myofibers at 4 (*n* = 15 Cre−, and 13 MRcko) (*n* = 9 untreated, and 8 spironolactone), 7 (*n* = 12 Cre−, and 14 MRcko) (*n* = 8 untreated, and 10 spironolactone), and 14 (*n* = 11 Cre−, and 13 MRcko) (*n* = 12 untreated, and 13 spironolactone) days post-injury in MRcko versus Cre− mouse littermates (left panel) and MR antagonist-treated and untreated wild-type littermate mice (right panel). The data are presented as mean ± SEM. ^**^p ≤ 0.01. **(B)** The distribution of centrally nucleated myofiber percentages was broken down by size in line graphs into small myofibers 75–600 μm^2^ and larger myofibers 600–3,600 μm^2^. The centrally nucleated myofiber size was analyzed at 4 (*n* = 15 Cre−, and 13 MRcko) (*n* = 9 untreated, and 8 spironolactone), 7 (*n* = 12 Cre−, and 14 MRcko) (*n* = 8 untreated, and 10 spironolactone), and 14 (*n* = 11 Cre−, and 13 MRcko) (*n* = 12 untreated, and 13 spironolactone) days post-injury. For size analysis, the following numbers of centrally nucleated myofibers were analyzed for each group and days post-injury: 2,653 ± 105 Cre− and 2,584 ± 161 MRcko at 4 days post-injury, 2,810 ± 166 Cre− and 2,818 ± 181 MRcko at 7 days post-injury, 3,103 ± 157 Cre− and 3,173 ± 193 MRcko at 14 days post-injury, 2,466 ± 167 untreated and 2,697 ± 167 spironolactone treated at 4 days post-injury, 2,465 ± 165 untreated and 2,629 ± 130 spironolactone treated at 7 days post-injury, and 2,730 ± 159 untreated and 3,019 ± 157 spironolactone treated at 14 days post-injury. **(C)** Bar graph showing representative myogenic factor and myofiber growth genes assessed in spironolactone treated (*n* = 5) and untreated (*n* = 5) littermates at 7 days post-injury. Beta-actin levels are used as a normalization control for each sample and fold-changes are normalized to the same untreated control. *Mstn*, myostatin; *Igf1*, insulin-like growth factor 1; *Myog*, myogenin; *Myod1*, myoblast determination protein 1. The data are presented as mean ± SEM. All data were analyzed using a Student’s *t*-test, ^*^p ≤ 0.05 and ^**^p ≤ 0.01 for the bins of fiber-sizes shown by the bracket.

To quantify an effect of MR signaling on myofiber growth, we measured the size of newly regenerated centrally nucleated myofibers in TAs from MRcko and spironolactone-treated mice compared to acutely injured controls at 4, 7, and 14 days post-injury. The process of measuring only centrally nucleated myofiber size excludes any uninjured regions of the muscle that could mask any phenotypic size differences of regenerating myofibers. The MRcko mice at 4 days post-injury had a trend (*p* = 0.0529) of increased average centrally nucleated myofiber size (439.11 ± 12.42 μm^2^) compared to Cre− mice (388.01 ± 20.80 μm^2^) (Figure 4A). The MRcko mice at 4 days post-injury had a lower percentage (*p* = 0.0276) of small centrally nucleated myofibers (52.91 ± 1.77%) for the 100–400 μm^2^ range compared to Cre− mice (60.29 ± 2.51%) and a higher percentage (*p* = 0.0398) of larger centrally nucleated myofibers (7.73 ± 0.72%) for the 700–1,200 μm^2^ range compared to Cre− mice (5.27 ± 0.86%) (Figure 4B). There was no difference in centrally nucleated myofiber size at 7 or 14 days post-injury in TAs from MRcko mice compared to Cre− littermates (Figures 4A,B).

In contrast to MRcko mice at 4 days post-injury, TAs from the spironolactone-treated mice had a trend (*p* = 0.0597) of decreased average centrally nucleated myofiber size (377.98 ± 26.14 μm^2^) compared to untreated mice (478.34 ± 40.14 μm^2^) (Figure 4A). The spironolactone-treated mice at 4 days post-injury had a higher percentage (*p* = 0.0217) of small centrally nucleated myofibers (23.62 ± 1.26%) for the 300–500 μm^2^ range compared to untreated mice (18.43 ± 1.54%) and a lower percentage (*p* = 0.0193) of larger centrally nucleated myofibers (7.26 ± 1.32%) for the 1,000–3,100 μm^2^ range compared to untreated mice (13.59 ± 1.95%) (Figure 4B). Moreover, the spironolactone-treated mice at 7 days post-injury had a significant decrease (*p* = 0.0055) in average centrally nucleated myofiber size (652.72 ± 18.99 μm^2^) compared to untreated mice (767.42 ± 32.38 μm^2^) (Figure 4A). The spironolactone-treated mice at 7 days post-injury had a higher percentage (*p* = 0.0177) of small centrally nucleated myofibers (46.54 ± 1.31%) for the 75–300 μm^2^ range compared to untreated mice (40.04 ± 2.22%) and a lower percentage (*p* = 0.0072) of larger centrally nucleated myofibers (28.63 ± 1.32%) for the 1,000–2,600 μm^2^ range compared to untreated mice (34.84 ± 1.53%) (Figure 4B). Additionally, the spironolactone-treated mice at 14 days post-injury had a significant decrease (*p* = 0.0091) in average centrally nucleated myofiber size (1143.24 ± 48.72 μm^2^) compared to untreated mice (1315.12 ± 33.91 μm^2^) (Figure 4A). The spironolactone-treated mice at 14 days post-injury had a higher percentage (*p* = 0.0021) of small centrally nucleated myofibers (38.52 ± 1.97%) for the 100–600 μm^2^ range compared to untreated mice (30.30 ± 1.25%) and a lower percentage (*p* = 0.0264) of larger centrally nucleated myofibers (24.85 ± 1.92%) for the 1,000–2,600 μm^2^ range compared to untreated mice (30.60 ± 1.43%) (Figure 4B). After we observed reduced myofiber size at 7 and 14 days post-injury resulting from spironolactone treatment, we evaluated the transcript levels of key myogenic genes and key regulators of myofiber growth. There were no differences in the levels of myoD (determination factor of myogenic lineage), myogenin (aids in fusion of muscle precursor cells with myofibers), myostatin (negatively regulates myofiber growth), or insulin-like growth factor 1 (positively regulates myofiber growth) in spironolactone-treated mice at 7 days post-injury compared to untreated (Figure 4C; Rodriguez et al., 2014; Sharples et al., 2015; Hernandez-Hernandez et al., 2017).

### MRcko Mice Have More CYP11B2 Infiltration and Spironolactone-Treated Mice Have More Fibrosis at 4 Days Post-injury

**Figure 5:**
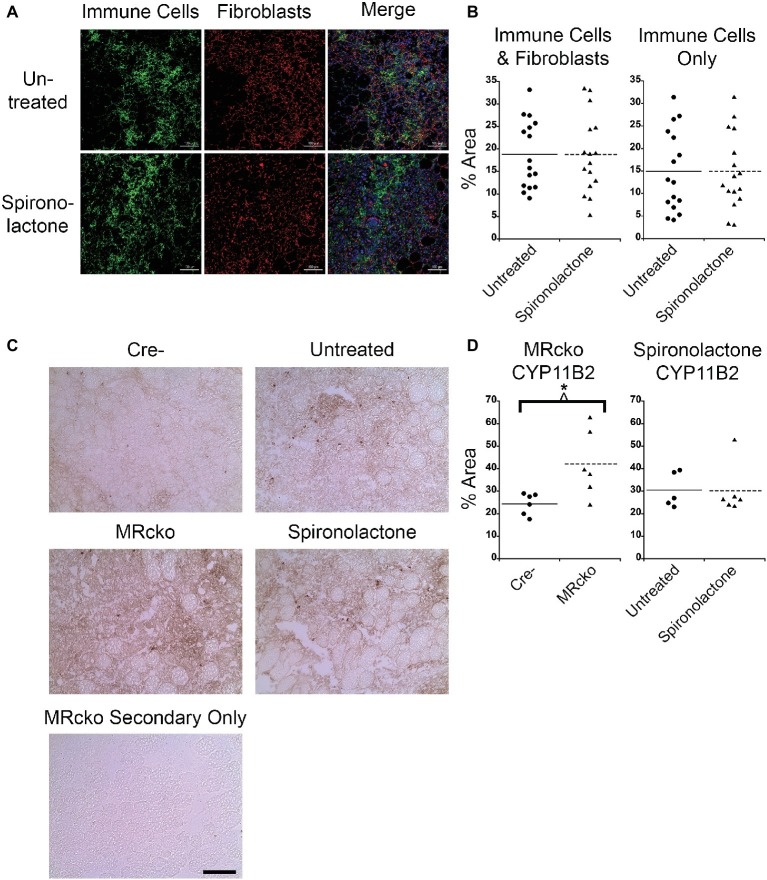
MRcko mice have increased CYP11B2 infiltration at 4 days post-injury. **(A)** Acutely injured *tibialis anterior* (TA) muscles of spironolactone treated and untreated mice at 4 days after acute injury were stained for CD11b to visualize myeloid immune cells (green), vimentin to visualize fibroblasts (red), and with DAPI to visualize nuclei (blue in merged image) and were imaged with confocal microscopy. Zoomed out images are shown to depict the areas of muscle infiltrated by immune cells (green dots) or fibroblasts (red dots) quantified in B. Scale bar = 100 μm. **(B)** Dot plots of spironolactone treated and untreated mouse (*n* = 16 spironolactone, and 16 untreated) TA muscle percent area at 4 days post-injury of immune cells and fibroblast infiltration together or percentage of myeloid immune cells infiltration alone. Means are shown by lines for each group in the dot plots. **(C)** CYP11B2 immunohistochemistry was analyzed at 4 (*n* = 6 Cre−, and 6 MRcko) (*n* = 5 untreated, and 6 spironolactone) days post-injury. Scale bar = 100 μm. **(D)** Dot plots of CYP11B2 percent infiltration at 4 days post-injury. Means are shown by lines for each group in the dot plots. All data was analyzed using a Student’s *t*-test. ^*^p ≤ 0.05.

After we observed a delay in myofiber growth post-injury in TAs from spironolactone-treated mice, we measured the muscle areas infiltrated by immune cells and fibroblasts because these cell types produce transforming growth factor β, a profibrotic growth factor and potent inhibitor of muscle regeneration (Taylor et al., 2011; Delaney et al., 2017). We stained spironolactone-treated mouse TA sections at 4 days post-injury with CD11b, a myeloid immune cell marker that is present at highest levels on monocytes and macrophages, and vimentin, a fibroblast marker (Zheng et al., 2015; Cheng et al., 2016; Tarbit et al., 2019). We saw no difference in the muscle area infiltrated by immune cells and fibroblasts or immune cells alone at 4 days post-injury from spironolactone treatment (Figures 5A,B), likely due to the wide variability in this measurement. Since we determined that immune cells produce CYP11B2 in acutely injured muscle, we quantified the muscle area infiltrated by CYP11B2-positive cells at 4 days post-injury in MRcko and spironolactone-treated mice. The MRcko mice at 4 days post-injury had a higher percentage (*p* = 0.0190) of CYP11B2 infiltration (42.13 ± 6.02%) compared to Cre− mice (24.44 ± 1.94%) (Figures 5C,D). However, there was no difference in percentage of CYP11B2 infiltration between spironolactone treated and untreated mice at 4 days post-injury.

**Figure 6:**
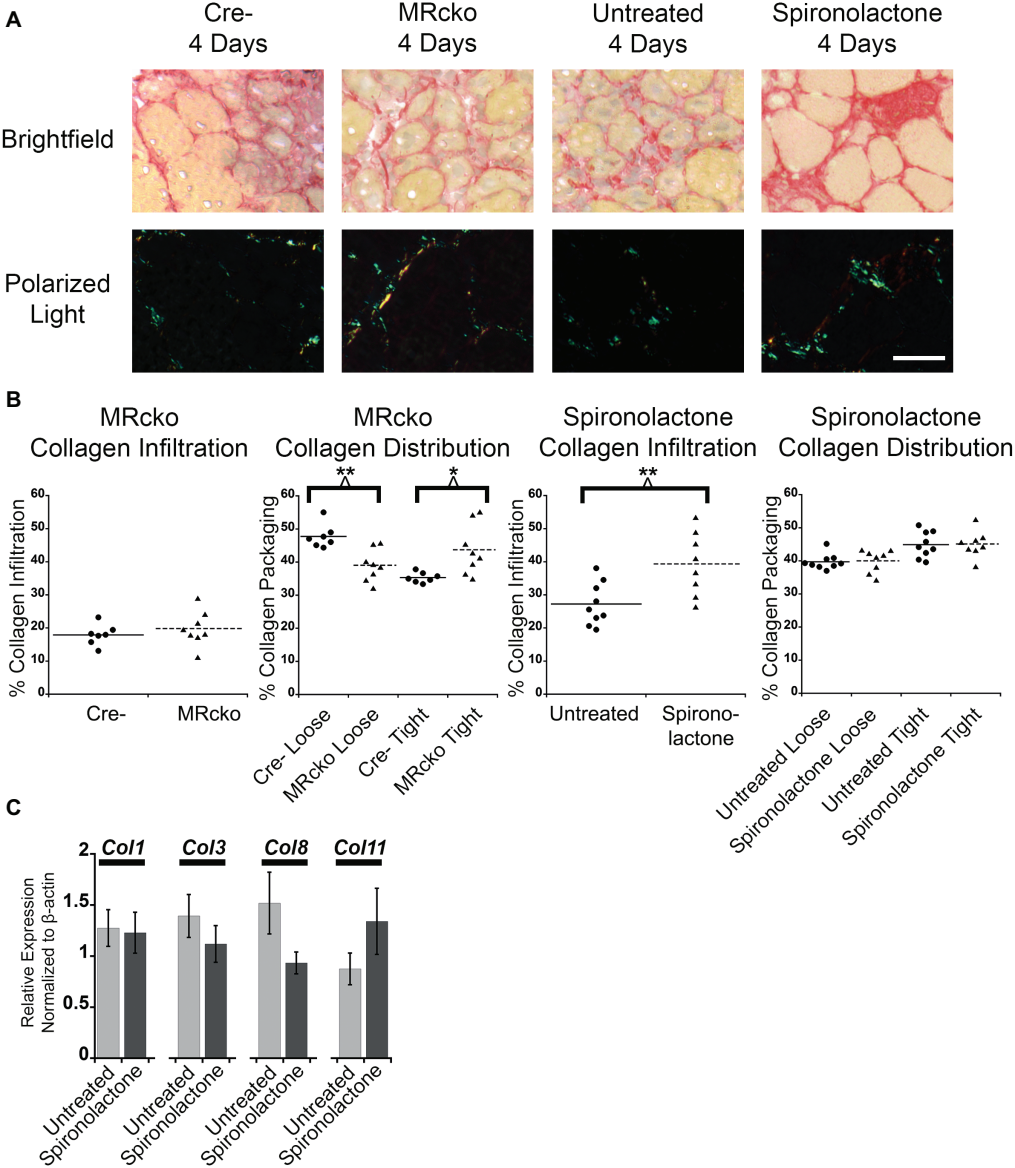
MRcko and MR antagonist treatment results in increased detrimental collagen infiltration. **(A)** Representative images of *tibialis anterior* muscle after picrosirius red staining under brightfield to quantify total collagen and polarized light to quantify collagen packaging. Loosely packaged collagen appears green and tightly packaged collagen appears red under polarized light. Scale bar = 40 μm **(B)** Dot plots of percent muscle area containing collagen infiltration and percent of loosely and tightly packaged collagen at 4 days after acute muscle injury for MRcko mice (*n* = 7 Cre−, and 8 MRcko) and spironolactone-treated mice (*n* = 8 untreated, 8 spironolactone) Means are shown by lines for each group in the dot plots. **(C)** Bar graph showing four representative collagen genes involved in fibrosis assessed in spironolactone treated (*n* = 5) and untreated (*n* = 5) littermates. Beta-actin levels were used as a normalization control. *Col1*, collagen 1 alpha 1; *Col3*, collagen 3 alpha 1; *Col8*, collagen 8 alpha 1; *Col11*, collagen 11, alpha 1. Data are presented as mean ± SEM. All data were analyzed using a Student’s *t*-test, ^*^p ≤ 0.05 and ^**^p ≤ 0.01.

To further evaluate fibrosis at 4 days post-injury, we stained MRcko and spironolactone-treated mouse TA sections with sirius red to visualize collagen infiltration. Under brightfield microscopy, total sirius red staining appears red, and under polarized light, the loosely packaged collagen appears green and the tightly packaged collagen appears red. The MRcko mice at 4 days post-injury did not have a difference in total collagen infiltration compared to Cre− controls. However, MRcko mice 4 days post-injury had a lower (*p* = 0.0017) percentage of green loosely packaged collagen (39.04 ± 1.70%) compared to Cre− mice (47.75 ± 1.35%) and a higher (*p* = 0.0132) percentage red tightly compacted collagen (43.71 ± 2.68%) compared to Cre− mice (35.32 ± 0.58%) (Figures 6A,B). There was substantially increased (*p* = 0.0074) collagen infiltration in spironolactone-treated mice at 4 days post-injury (39.34 ± 3.38%) compared to untreated mice (27.24 ± 2.14%), but there was no difference in collagen compaction compared to untreated mice (Figures 6A,B).

After we observed the increase in total collagen infiltration from spironolactone treatment, we evaluated the levels of collagen transcripts that regulate fibrosis. There was no difference in the level of collagen type 1, 3, 8, or 11 in spironolactone-treated mice at 4 days post-injury compared to untreated (Figure 6C).

## Discussion

Here we show for the first time that MR signaling is a critical regulator of acute injury responses in skeletal muscle. We demonstrate that myeloid immune cells in acutely injured skeletal muscle contain CYP11B2 aldosterone synthase that has the potential to regulate MR present in multiple cell types in the microenvironment. MRcko muscles at 4 days post-injury had a larger area of CYP11B2 enzyme localization compared to Cre− mice. It is well known immune cell infiltration into acutely injured skeletal muscle peaks around 4 days after acute injury, so the increase in CYP11B2 at that time point is likely due to myeloid immune cell infiltration (Bentzinger et al., 2013; Chadwick et al., 2016; Juban and Chazaud, 2017; Tidball, 2017). The increase in CYP11B2 in MRcko muscles at 4 days after injury compared to Cre− muscles suggests that ablation of the myofiber MR may remove part of a negative feedback loop to reduce production of aldosterone synthase. However, there was no difference in CYP11B2 localization from spironolactone treatment at 4 days post-injury compared to untreated. Together, these data indicate that aldosterone likely produced by these CYP11B2 containing immune cells in damaged muscle may stimulate the MR in other infiltrating immune cells to increase CYP11B2 levels in a positive feedback loop. When enough aldosterone is produced by immune cells, the subsequent binding of aldosterone to the MR of nearby myofibers, may activate a negative feedback loop that reduces the levels of CYP11B2 aldosterone synthase in immune cells.

In the current study, we defined the effect of MR signaling post-injury on muscle damage and recovery through the use of MR knockout in myofibers and MR antagonist treatment that inactivates MR signaling in all cell types of the muscle microenvironment. There were more damaged myofibers measured at 4 days post-injury both after ablation of MR signaling in myofibers and after pharmacological inhibition in all cell types of the muscle microenvironment. While there were more damaged fibers from blocking MR signaling in both models, there was no difference in cumulative damage measured by the percentage of centrally nucleated fibers at any time point post-injury for either model. These data indicate that blocking MR signaling stabilized damaged myofibers at 4 days post-injury but did not have an effect on total muscle damage. These data further support that MR antagonist treatment is protective for fragile myofibers, as we have shown in muscular dystrophy models (Rafael-Fortney et al., 2011; Lowe et al., 2016; Chadwick et al., 2017a; Hauck et al., 2019). The spironolactone treated and untreated mice at 7 and 14 days post-injection had a decrease in cumulative damage compared to the MRcko and Cre− mice. This decrease in cumulative damage was likely due to variability in the acute injury technique performed at different times in the cohorts of mice because the spironolactone treated and untreated mice appeared to have more undamaged regions of the TA at 7 and 14 days post-injection compared to the MRcko and Cre− mice.

The increase in myofiber size for the MRcko mice at 4 days post-injury was likely related to stabilization of damaged myofibers. Through the method of measuring centrally nucleated myofiber size in an unbiased manner, damaged myofibers that have been infiltrated with immune cells are counted as centrally nucleated myofibers by the software because they have nuclei in the center of the damaged myofiber. The stabilization of degenerating myofibers in MRcko mice likely resulted in the increase in centrally nucleated myofiber size at 4 days post-injury because damaged myofibers are larger than newly formed myofibers. However, there was no difference in centrally nucleated myofiber size for MRcko mice at 7 or 14 days post-injury. Spironolactone treatment also stabilized damaged myofibers at 4 days post-injury, but the decrease in centrally nucleated myofiber size at 4 days post-injury continued through 7 and 14 days post-injury when damaged myofibers no longer persist, supporting an effect of MR signaling on myofiber growth. These data indicate that spironolactone treatment inhibits MR signaling in cell types in the muscle microenvironment other than myofibers that promote myofiber growth and are activated before 7 days post-injury. Future experiments will be needed to determine whether MR signaling in satellite cells contributes to their activation, proliferation, mobility or fusion. Alternatively, MR signaling in infiltrating immune cells or fibroblasts may have downstream effects on myofiber growth. There was no difference in levels of myogenic factor or growth regulation transcripts at 7 days post-injury in spironolactone-treated compared to untreated littermates, suggesting that more complex mechanisms likely underlie the mildly delayed muscle regeneration post-injury. Although it is also possible that spironolactone’s function as a potassium-sparing diuretic may contribute to the observed effects on muscle, these mechanisms are unlikely. Blood pressure changes are absent in previous studies of normotensive dystrophic mice using the stronger blood pressure lowering angiotensin converting enzyme inhibitor drugs (Lowe et al., 2015). Additionally, spironolactone is used routinely in normotensive individuals with mild conditions such as unwanted facial hair, demonstrating that its diuretic activity also does not clinically cause complications in normotensive individuals (van Zuuren et al., 2015). It is also possible that spironolactone’s low-level off-target effects on androgen receptors could affect skeletal muscle regeneration, but the increase in aldosterone synthase localization in muscles from MRcko, but not spironolactone-treated mice suggest specific targeting of MR in regenerating muscle. In this study, we sought to define the role of the myofiber MR in the normal muscle damage response. However, future experiments may identify a greater role for MR signaling in extreme damage responses after repeated acute muscle injuries or in the muscle damage response in aged sarcopenic animals (Dessem and Lovering, 2011; Desguerre et al., 2012; Pessina et al., 2014; Myers et al., 2019).

Inhibition of MR signaling increased the stiffness of the acutely injured muscles by increasing collagen compaction and infiltration. The MRcko mice at 4 days post-injury had more tightly bound collagen fibrils compared to the Cre− mice. This increase in collagen bundling may be related to the stabilization of damaged myofibers in MRcko mice. Spironolactone treatment increased total collagen infiltration 4 days post-injury, which may also be related to stabilization of damaged myofibers or from inactivating MR signaling in another cell type in the acutely injured muscle. Fibrosis causes cellular barriers that can impede the ability of muscle precursor cells to regenerate damaged muscle, so the increase in collagen infiltration may be partially responsible for the decrease in myofiber size from spironolactone treatment (Petrosino et al., 2019). To determine if spironolactone treatment was regulating fibrosis at 4 days post-injury, we evaluated the levels of fibrotic collagen transcripts. We have previously reported that treatment with the profibrotic MR agonist aldosterone in cultured myotubes increases expression of collagen type 8, an enhancer of collagen of secretion, and collagen type 11, a regulator of collagen fibril assembly (Shuttleworth, 1997; Wenstrup et al., 2011; Lopes et al., 2013; Skrbic et al., 2015; Vazquez-Villa et al., 2015; Chadwick et al., 2017b). Additionally, collagen types 1 and 3 were evaluated because they make up the majority of fibrotic lesions (Coelho and McCulloch, 2016; Karsdal et al., 2017; Mahdy, 2019). There was no difference in levels of collagen transcripts at 4 days post-injury compared to untreated. These data indicate that either collagen transcripts are regulated at an earlier time point post-injury or that collagen protein stability is altered from spironolactone treatment to enhance collagen infiltration post-injury.

The efficacy of MR antagonist treatment in muscular dystrophy likely results from inhibiting chronically activated MR signaling. Since disease pathogenesis often results from chronic activation of normal biological processes, it is not surprising that MR signaling contributes to normal muscle regeneration. CYP11B2 was present in immune cells in damaged muscle at 4 days post-injury supporting that MR hormonal regulation is a contributing factor to the muscle damage response for both acute and chronic muscle injury. In this study, we showed for the first time that MR antagonist treatment delayed muscle myofiber growth post-injury. Therefore, patients who are actively taking MR antagonists at the time of severe muscle injury should consider halting the use of this drug for 1–2 weeks post-injury. MR antagonists are not the only pharmacological treatment known to slow muscle recovery from acute muscle injury; treatment with cyclooxygenase 2 specific inhibitors delays recovery from acute muscle injury through inhibition of the immune response and muscle stem cells (Bondesen et al., 2004; Mendias et al., 2004; Novak et al., 2009). While MR antagonist treatment slows muscle recovery from a severe acute muscle injury, MR antagonist treatment may be beneficial for professional athletes and military personnel who regularly receive mild to moderate forms of acute muscle injury from repeated, intense exercise (Nindl et al., 2002; Henning et al., 2011; Brisswalter and Nosaka, 2013; Peake et al., 2017). Muscle damage from repeated, intense exercise causes low athletic performance from loss of muscle strength, ion imbalance, soreness, swelling, and decreased range of motion (Ihsan et al., 2016; Hostrup and Bangsbo, 2017; Peake et al., 2017). Prophylactic treatment with non-steroidal anti-inflammatory drugs like ibuprofen is commonly used for professional athletes and military personnel to deal with the side effects of repeated intense exercise, but prolonged use of these drugs can lead to a propensity for infection (Bryant et al., 2017). Prophylactic MR antagonist treatment for repeated, intense exercise may stabilize damaged myofibers and reduce immune cell and fibroblast infiltration into damaged muscle in a similar manner as muscular dystrophy patients without compromising the normal immune response to pathogens (Rafael-Fortney et al., 2011; Lowe et al., 2016; Bryant et al., 2017; Chadwick et al., 2017a; Hauck et al., 2019). Future work should be done to determine if MR antagonist treatment reduces recovery time from repeated, intense exercise or whether modulation of MR signaling can accelerate the muscle repair process.

## Data Availability Statement

All datasets generated for this study are included in the manuscript/supplementary files.

## Ethics Statement

The animal study was reviewed and approved by Institutional Animal Care and Use Committee of The Ohio State University.

## Author Contributions

JH and JR-F designed the experiments. JH, JL, NR, MP, ZH, SS, and JP performed the experiments. CG-S and EG-S provided required reagents for the experiments. JH and JL performed statistical analyses. JH, FA, and JR-F interpreted the data. JH and JR-F wrote the manuscript. All authors read, edited, and approved the manuscript for submission.

### Conflict of Interest

The authors declare that the research was conducted in the absence of any commercial or financial relationships that could be construed as a potential conflict of interest.

## Abbreviations:

CD11b: Cluster of differentiation 11b, a myeloid immune cell marker
Cre−: Mineraocorticiod receptor floxed mice without Cre recombinase
CYP11B2: Cytochrome P450 group 11, subgroup B, gene2; aldosterone synthase enzyme
DAPI: 4′,6-Diamidino-2-phenylindole; stains nuclei blue
MCK-Cre: Muscle creatine kinase promoter driven Cre recombinase
MR: Mineralocorticoid receptor
MRcko: Mineralocorticoid receptor conditional knockout
PBS: Phosphate buffered saline
qPCR: Quantitative polymerase chain reaction
SMASH: Semi-automatic muscle analysis using segmentation of histology
TA: *Tibialis anterior* muscle.
